# Can Physical Activity Influence Human Gut Microbiota Composition Independently of Diet? A Systematic Review

**DOI:** 10.3390/nu13061890

**Published:** 2021-05-31

**Authors:** Barbara Dorelli, Francesca Gallè, Corrado De Vito, Guglielmo Duranti, Matteo Iachini, Matteo Zaccarin, Jacopo Preziosi Standoli, Roberta Ceci, Ferdinando Romano, Giorgio Liguori, Vincenzo Romano Spica, Stefania Sabatini, Federica Valeriani, Maria Sofia Cattaruzza

**Affiliations:** 1 Department of Public Health and Infectious Diseases, Sapienza University of Rome, 00185 Roma, Italy; barbara.dorelli@uniroma1.it (B.D.); corrado.devito@uniroma1.it (C.D.V.); matteo.iachini@uniroma1.it (M.I.); zaccarin.1674425@studenti.uniroma1.it (M.Z.); preziosistandoli@gmail.com (J.P.S.); ferdinando.romano@uniroma1.it (F.R.); mariasofia.cattaruzza@uniroma1.it (M.S.C.); 2 Department of Movement Sciences and Wellbeing, University of Naples “Parthenope”, 80133 Napoli, Italy; francesca.galle@uniparthenope.it (F.G.); giorgio.liguori@uniparthenope.it (G.L.); 3 Department of Movement, Human, and Health Sciences, University of Rome “Foro Italico”, 00135 Roma, Italy; roberta.ceci@uniroma4.it (R.C.); vincenzo.romanospica@uniroma4.it (V.R.S.); stefania.sabatini@uniroma4.it (S.S.)

**Keywords:** physical activity, diet, microbiota, human, gut, healthy, biodiversity

## Abstract

Evidence suggests that physical activity (PA) influences the human gut microbiota composition, but its role is unclear because of dietary interference. The aim of this review is to clarify this issue from this new perspective in healthy individuals. Articles analyzing intestinal microbiota from fecal samples by 16S rRNA amplicon sequencing were selected by searching the electronic databases PubMed, Scopus, and Web of Science until December 2020. For each study, methodological quality was assessed, and results about microbiota biodiversity indices, phylum and genus composition, and information on PA and diet were considered. From 997 potentially relevant articles, 10 met the inclusion criteria and were analyzed. Five studies involved athletes, three were performed on active people classified on the basis of habitual PA level, and two among sedentary subjects undergoing exercise interventions. The majority of the studies reported higher variability and prevalence of the phylum Firmicutes (genera *Ruminococcaceae* or *Fecalibacteria*) in active compared to inactive individuals, especially in athletes. The assessment of diet as a possible confounder of PA/exercise effects was completed only in four studies. They reported a similar abundance of *Lachnospiraceae*, *Paraprevotellaceae*, *Ruminococcaceae*, and *Veillonellaceae*, which are involved in metabolic, protective, structural, and histological functions. Further studies are needed to confirm these findings.

## 1. Introduction

The human gut microbiota has been defined as the entire collection of microbes (bacteria, archaea, eukarya, and viruses) living as a complex ecosystem in our gastrointestinal tract, coevolved with humankind [1]. The recent introduction of next-generation sequencing systems and the increasing number of metagenomics analyses have allowed scientists to increase our knowledge regarding the composition and diversity of these populations of microorganisms by characterizing the so-called gut microbiome [2]. As for bacteria, the main taxa represented are, in different proportions, Firmicutes, Bacteroides, Cyanobacteria, Proteobacteria, Fusobacteria, Actinobacteria, and Verrucomicrobia, with the first two phyla representing about 90% of the bacterial flora in the gut [3,4]. Current evidence shows that these phyla are not only involved in a series of local processes such as mucosal homeostasis and maintenance of epithelial integrity, as well as protection from pathogenic microorganisms, biosynthesis, and absorption of nutrients, but they also interact with the immune system and with the nervous system, being part of the recognized brain–gut axis [1,4,5]. The composition and diversity of intestinal microbiota have been associated with several chronic diseases, including colorectal cancer, metabolic, autoimmune, and allergic diseases, and neurological disorders [6,7,8,9,10,11,12,13]. Gut microbes can affect the homeostasis of the host by producing vitamins, amino acids, and short-chain fatty acids (SCFA) starting from food components [14]. Therefore, by providing substrates for microbial metabolism, diet has a fundamental role in determining gut microbiota composition and diversity [15,16].

Recent studies have also suggested the possible influence of physical activity (PA) on gut microbiota composition [17,18,19,20,21]. PA—including leisure activities, planned exercise, and sport—has been proven to be a key component of a healthy lifestyle and a fundamental tool in the prevention and treatment of several chronic diseases [22]. Therefore, the definition of its possible effects on the intestinal microbiota might reveal further mechanisms by which PA acts, interacting with or independently of diet, on human health. Evidence suggests that some microbial phyla and genera whose abundance was found to be exercise-related, especially SCFA producers, may have a role in maintaining intestinal epithelial homeostasis and increasing mucus thickness, in improving host metabolic immune status, and in modulating the gut–brain axis, reducing, among other things, neuroinflammation and mental fatigue [17,18,19,20,21,23,24,25]. Nevertheless, exercise-induced stress may be correlated with changes in the gastrointestinal microbiota composition [24]. However, the majority of the available studies focused on this issue are based on animal models or performed among nonhealthy individuals [19,20]. In addition, studies performed in humans so far have had a heterogeneous design and considered different forms, doses, and duration of exercise, which did not allow drawing clear conclusions [20,21,23]. Furthermore, the interaction between PA and diet composition and their respective influence on gut microbiota are not always characterized [18,21,23]. Some authors have tried to systematically review the available evidence concerning PA effects on gut microbiome composition in healthy humans by using stringent selection criteria [21,24,25,26]. They identified some PA-related effects on bacterial abundance and diversity indices. As research in this field is rapidly growing, there is a need to update the findings on this issue. Furthermore, although studies based on dietary interventions were excluded by these reviews, the role of diet as a possible confounding factor for the identified PA effects was not systematically appraised.

The aim of this systematic review is to better understand whether and how PA can influence the human gut microbiota composition independently of diet. To this aim, controlled studies exploring the relationship between PA and the human microbiome in healthy individuals were evaluated. The findings of these studies were examined in light of the possible confounding effect of dietary factors.

## 2. Materials and Methods

**Figure 1:**
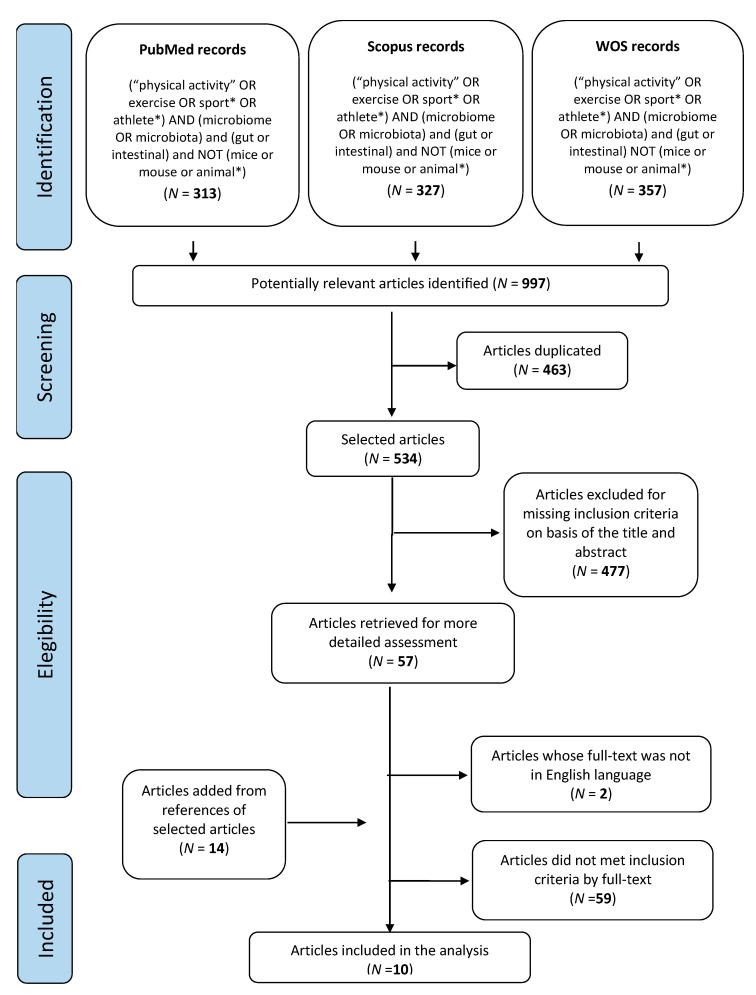
PRISMA flow diagram of the systematic review process.

This systematic review was performed adhering to the Preferred Reporting Items for Systematic Reviews and Meta-Analysis statement (PRISMA) and was registered with the International Prospective Register of Systematic Reviews (PROSPERO registration number: CRD42020170277) [27]. The following databases were searched: PubMed, Scopus, and Web of Science. Since the aims of this review were different from those of previously published systematic reviews, these were not used as a starting point, and all the available literature published until 31 December 2020 was considered. To identify relevant studies, the string “(physical activity OR exercise OR sport* OR athlete*) AND (microbiome OR microbiota) AND (gut or intestinal) and NOT (mice or mouse or animal*)” was used, according to the research criteria of each database (Figure 1). After reviewing each title and abstract, the authors reviewed the list of references for citations that could have been missed by the initial search. The inclusion criteria were the following: any study design with a control group; healthy humans as subjects, without gender or age limitations; analysis of intestinal microbiota from fecal samples and by 16S rRNA amplicon sequencing; English language. Studies performed in vitro or involving animals, involving subjects with any pathological status, or performed without controls, those examining microbiota through intestinal biopsy or other biological samples than fecal ones and based on analytical methods other than 16S rRNA amplicon sequencing, and those published in non-English languages were excluded.

For each study, the main results regarding gut microbiota biodiversity indices and composition at phylum and genus levels were considered. The studies included in this systematic review measured alpha diversity, which represents diversity within a sample. In calculating alpha diversity, various metrics (e.g., Shannon index, Chao1) were used, both as “richness” and “evenness or equitability”. Moreover, beta-diversity indices were used to evaluate the different structure of the communities between samples, both considering samples’ phylogeny (weighted UniFrac) and evaluating the presence/absence of genera in the samples (unweighted UniFrac). Any reference to the dietary pattern of participants and its possible effects on microbiome composition was also examined. The included studies were assessed for methodological quality using the JBI Critical Appraisal Checklist for Analytical Cross-Sectional Studies (2017) and Cochrane Risk of Bias Tool for Randomized Controlled Trials [28,29,30].

## 3. Results

### 3.1. Characteristics of Selected Studies

From a total of 997 references which were potentially relevant, 534 articles plus another 14 titles identified from other articles were examined for eligibility (Figure 1). Ten articles [31,32,33,34,35,36,37,38,39,40] met all the inclusion criteria and were included in the review (Table 1). All the studies were performed in the last decade.

MET: metabolic equivalents; FFQ: Food Frequency Questionnaire; USDA: United States Department of Agriculture. Four studies were carried out in Europe [31,32,34,35], three in Asian countries [36,37,40], and three in the USA [33,38,39]. Eight of these [31,32,33,35,36,37,38,39] reported cross-sectional studies, while two [34,40] regarded clinical trials. The majority of the studies involved only male subjects [31,33,36,37,40]; one included only females [32], and three examined both genders [34,35,38], while one did not specify participants’ gender [39]. Five of the cross-sectional studies analyzed subjects practicing sports at professional or recreational level [31,36,37,39]; three articles took into account the weekly exercise levels of the participants [32,35,38]. The longitudinal studies analyzed the effects of aerobic and resistance training performed for 8 and 5 weeks on previously sedentary subjects [34,40]. All the cross-sectional studies had a sufficient quality [31,32,33,35,36,37,38,39], while longitudinal trials showed a fair quality [34,40]. The overall risk of bias judgments is reported in Tables S1 and S2.

Since five of the selected studies involved athletes [31,33,36,37,39], while the other five were performed on people classified on the basis of their habitual PA level [32,35,38] or sedentary subjects undergoing exercise interventions [34,40], the results of these different categories of individuals compared to their inactive controls have been reported separately in Table 2 and Table 3. 

### 3.2. Studies Involving Athletes

As for the gut microbiota biodiversity of the examined athletes, three studies showed higher values of Shannon index [31,33,36] compared to controls, while one of them [33] reported a lower Unifrac distance and another reported no difference in beta diversity between athletes and controls [37]. The analysis of microbiota composition in the athletes revealed a higher prevalence of genera and species belonging to the phylum Firmicutes in all five studies [31,33,36,37,39], although Jang et al. reported a lower abundance of the genera *Blautia*, *Leuconostoc*, and *Weissella* in runners’ fecal samples compared to controls [37]. Three studies reported a decrease in the phylum Bacteroidetes [36] or in genera belonging to this phylum [33,37]; the study by Clarke et al. reported an increase in the genus *Prevotella* among rugby players [33]. As for Actinobacteria, Jung et al. found a lower abundance of the genus Bifidobacterium in body builders [37]. The same study registered, for Proteobacteria, an increased abundance of *Sutterella* and *Haemophilus* and decreased abundance of *Parasutterella*, *Acinetobacter*, and *Enterobacter* in athletes compared to controls [37]. Two studies found significantly higher concentrations of the genus *Akkermansia* in athletes [31,33].

### 3.3. Studies Performed in Non-Athlete Populations

Among the selected studies performed in non-athlete populations, three reported higher Shannon indices [34,38] and beta diversity [35] in active people compared to the inactive ones, while three did not find any differences in alpha diversity [32,34,40]. Two studies reported higher levels of the Firmicutes phylum or genera in active individuals [32,38], while two other studies registered a decrease in *Megasphaera*, *Lachnobacterium*, *Dialister*, and *C. difficile* concentration related to PA [35,40]. As for Bacteroidetes, two studies found a lower abundance of *Barnesiellaceae* and *Odoribacteriaceae* [32] and of *Paraprevotella* [35] in active people. No differences between active and inactive subjects were shown in the selected studies for both Actinobacteria and Proteobacteria phyla. Only Bressa et al. found a higher abundance of *Akkermansia* in active individuals [32].

### 3.4. Diet-Related Outcomes

All the studies analyzed the dietary habits of participants through questionnaires [31,32,33,34,35,36,37,38,39,40]. However, the investigation tools differed among these studies; some of them evaluated nutrient or food group intake [32,36,38], while one assessed adherence to Mediterranean diet [35]; three of them evaluated the use of dietary supplements [33,34,39], and only one controlled the diet for whey protein supplementation during the intervention. The use of dietary data was also different: The control of diet as a possible confounder of PA/exercise effect was performed in four studies [32,35,38,39], while an analysis of the correlation of gut microbiome diversity and composition with dietary features was carried out in another four [31,33,36,37]; two studies evaluated the results in light of dietary differences among the study groups [34,40]. Furthermore, the results of the studies which assessed the association of gut microbiome diversity and composition with dietary features were inconsistent. Barton et al. [31] reported a higher amount of microbial-derived SCFAs in athletes, some of which were associated with dietary components and with gut microbial diversity or the abundance of specific genera. Clarke et al. [33] found an enhanced gut microbiota diversity related to protein consumption and exercise in athletes, and Cronin [34] found the same with regard to the bacterial component. By contrast, Jang [37] reported a lower microbiome diversity and lower SCFA-producing bacteria in aerobic and resistance athletes, respectively, in association with a high-protein diet. No associations with diet were found by Bressa et al. [32], Han et al. [36], or Scheiman et al. [39], while Taniguchi et al. [40] showed that diet and physical characteristics, but not exercise, were associated with gut microbiome variability; the study by Gallè et al. [35] did not find any significant association between microbiome diversity and diet, nor between microbiome diversity and PA level. On the other hand, Manor et al. [38] reported a robust relationship between PA and microbiome diversity that was independent of major dietary factors.

**Figure 2:**
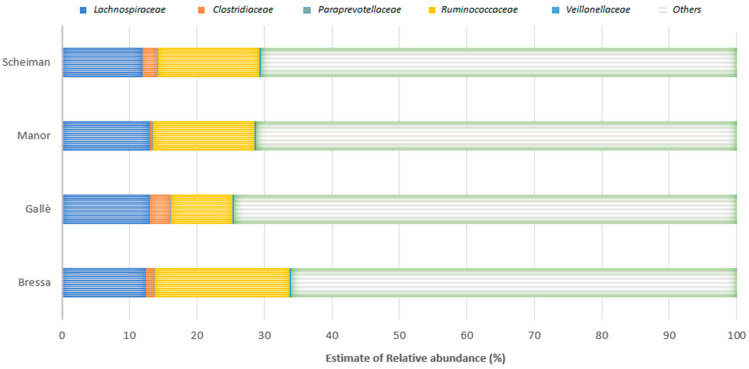
Main results reported by the four investigations that controlled PA effects for diet (Bressa et al., Gallè et al., Manor et al., Scheiman et al.) [32,35,38,39]. Results were expressed as values of bacterial family relative abundance/total number of sequences in the group * 100, rounded to the nearest integer.

The four studies that performed control of diet as a possible confounder of the PA/exercise effect [32,35,38,39] reported associations between PA and five bacterial families (*Lachnospiraceae*, *Clostridiaceae*, *Paraprevotellaceae*, *Ruminococcaceae*, and *Veillonellaceae*) (Figure 2). The relative abundance of these bacterial families was comparable in the different studies, with the only exception of *Clostridiaceae* (Figure 2).

## 4. Discussion

### 4.1. Focus and Novelty of This Review

This review was aimed at evaluating the role that PA may play in determining gut microbiota composition in healthy humans, trying to distinguish its effects from those of diet. The analysis of the controlled studies selected in this review highlighted some differences in bacterial variability and abundance between active and inactive people. In particular, higher variability and higher abundance of Firmicutes was shown in active healthy adults. Recent studies have suggested that higher levels of PA and cardiorespiratory fitness are associated with higher microbial diversity in the gut and with the abundance of some phyla and certain short-chain fatty acid producers in humans [17,18,19,20,41]. Previous reviews have tried to address this, but they did not reach clear conclusions due to the paucity and heterogeneity of the available studies on humans [21,23]. Since the design of the study, the populations examined, the type of PA considered, and the assessment methods are different in the literature, it is difficult to draw definite conclusions in this field. In their recent review, Tzemah Shahar et al. tried to characterize the role of PA in humans by exclusively analyzing studies reporting PA intervention with a duration of at least 5 weeks [26]. Aya et al., by contrast, included cross-sectional and longitudinal studies [25]. However, neither review evaluated these studies in light of how the relationship between PA and diet was considered.

Since the gut microbiota may be influenced by several factors, such as diet or disease, it is important to characterize the possible role of PA, excluding the effects of potential confounders. Therefore, while planning this review, we tried to choose eligibility criteria that could have allowed us to obtain a selection of comparable studies. To this end, we considered only controlled studies on healthy humans and evaluated their findings with regard to diet, assumed as the main possible confounder. Furthermore, in the analysis of the results, we separated those obtained from athlete groups and those coming from the general population to detect possible differences related to sport practice, active lifestyle, and sedentarism.

However, even with these limitations, the selected studies showed heterogeneous results regarding microbiome variability and composition, as well as for diet-related outcomes. This is probably due to the differences among the studies, which employed different measures and were conducted on samples from different geographical areas, practicing different sports or with levels of exercise, and with different age.

### 4.2. Findings and Comparison to Other Research

As regards microbial variability, six out of the ten studies found higher values in active people [31,33,34,35,38]. In addition, the study of Bressa et al. reported an inverse association between sedentary parameters and microbiota richness, suggesting that the pattern of exercise, such as breaks in sedentary time which help to avoid long periods of inactivity during daily routine, may induce changes in gut microbiota composition [32]. However, two of these [34,35] did not report any differences when using other variability indices, and another found a lower dissimilarity in athletes compared to controls [33]. These different results may be attributed to the different measures adopted to evaluate microbiota variability and are consistent with those of Tzemah Shahar et al. [26]. Interestingly, the studies which showed higher variability were performed in young adult groups [31,33,34,35,38]. The study by Taniguchi et al., which did not register different levels of variability, involved older adults [40]. It is possible that the different age classes played a role in determining these results. In fact, compared with young adults, the elderly has a different digestive physiology, characterized by a reduction in transit and production of digestive secretions, which could explain the changes in the fecal microbiota associated with advancing age [41,42,43]. In contrast to this, Jang et al. did not find a significant microbiota diversity between young athletes and healthy controls. However, as the authors stated, the inadequate intake of carbohydrates and dietary fiber associated with a high-protein diet observed in the athletes might have counteracted the beneficial effects of exercise on gut microbiota diversity. This unbalanced diet may be the cause of the inconsistency between these results and those of Clarke et al. and Cronin et al., who reported a higher microbial diversity in rugby athlete/exercise groups in relation to protein consumption [33,34]. Even analyzing a sample of rugby players, Barton et al. confirmed the enhancement of microbial diversity in professional athletes associated with extreme PA and dietary adaptations, such as increased protein intake, in comparison to sedentary individuals. Athletes also showed increased metabolic pathways and fecal metabolite production compared to controls [31].

As reported by other authors [24,26], several differences between athletes/active and inactive people were reported at the phylum and genus level, even by studies which did not detect significant variations in general variability measures. The majority of the studies found a higher abundance of the phylum Firmicutes or correspondent genera [31,32,33,34,35,36,37,38,39] and a lower prevalence of Bacteroidetes or related genera [32,33,35,36,37] in more active people. Contrarily to these results, a lower prevalence of *C. difficile* was detected in the exercise group by Taniguchi et al. [40], while a higher prevalence of the *Prevotella* genus was found in rugby players by Clarke et al. [33]. The Firmicutes/Bacteroidetes ratio has been found to be associated with several factors that can influence their balance in the gut. Age, gender, therapies, and diet may in fact favor one of these phyla, leading to dysbiosis, which can in turn allow the development of disease. In particular, an increased amount of Firmicutes compared to Bacteroidetes has been related to the pathophysiology of intestinal, metabolic, and central nervous system related disorders [43]. However, since the increase in some Firmicutes genera cannot necessarily be negative for the host, the findings of this review do not necessarily indicate that exercise may be detrimental to gut microbiome composition and human health. In fact, Firmicutes genera such as *Ruminococcaceae* or *Fecalibacteria*, which were reported to be higher among active participants in the studies by Clarke, Jung, and Han, have been shown to be beneficial for health and were associated with healthy status and lifestyle [33,36,37]. At the same time, the genus *Megasphaera*, which was reported to be lower in active individuals by Gallè et al., and the genus *Bacteroides*, whose decrease was identified by Clarke and Jang, were associated with a disease status [38]. Interestingly, among *Verrucomicrobia*, an increase in the genus *Akkermansia* was found among active people in three studies [31,32,33]. The role of this intestinal symbiont in host health has been widely shown [44]. In particular, the levels of *A. muciniphila* have been demonstrated to be negatively correlated with some diseases, included inflammatory bowel disease, obesity, and diabetes [45]; its activity in increasing intestinal mucus thickness, gut barrier, and immune signaling functions, and its role as an SCFA producer have made this species a promising candidate for next-generation probiotics. Other findings at genus level were study-specific and accounted for differences among athletes practicing different types of sport.

In conclusion, the results of this systematic review indicate that PA can increase the abundance of health-promoting bacteria in the intestinal microbiota, hindering some negative genera. In particular, a higher variability and abundance of Firmicutes was reported in the majority of the studies comparing athletes and sedentary people, while these findings were less robust in the studies performed in the general population. This could be related to the different volume of exercise between athletes and non-athletes and may suggest the importance of the volume of PA in determining gut microbiota composition. However, it should be noted that the practice of sport can be associated with specific food/nutrient intakes, which can favor specific microbial populations [21,24,26]. Therefore, it may be difficult to disentangle the effects of PA/exercise and diet. In our analysis, only four cross-sectional investigations adjusted their results for diet [32,35,38,39]. Two of these studies refer to the lack of significant correlations after controlling for confounders, while Manor et al. confirmed a significant association between PA and microbiome diversity after adjusting for dietary factors [38]. The study by Scheiman et al. found a diet-independent increase in the genus *Veillonella*, suggesting the possibility that systemic lactate resulting from muscle activity during exercise may enter the gastrointestinal lumen and become metabolized by this genus, providing a selective advantage for gut colonization by lactate-metabolizing organisms [39]. Interestingly, the relationship between PA levels and *Veillonella* abundance in the gut was also reported by Manor et al. [38]. Moreover, it should be noted that these four studies highlighted similar findings regarding the abundance of bacterial families (*Lachnospiraceae*, *Paraprevotellaceae*, *Ruminococcaceae*, and *Veillonellaceae*) that are involved in several function and different pathways, including metabolic, protective, structural, and histological functions [32,35,38,39]. *Lachnospiraceae* include genera such as *Coprococcus*, a butyrate-producing genus, which promote some exercise-related health effects. Moreover, *Lachnospira* species are known to produce anti-inflammatory short-acid butyrate [46]. Furthermore, the families *Lachnospiraceae* and *Ruminococcaceae* can be anticorrelated in gut microbiota because they overlap as an ecological niche. Indeed, these families can respond to similar diets, such as high fiber or probiotics [47]. However, both can be involved in fiber degradation and butyrate production [36,45]. *Clostridiaceae* are associated with an increase in fecal butyrate production among physically fit participants and involved in these pathways [48,49]. However, this bacterial family can be influenced by intake of a high-fat diet [50]. *Veillonellaceae* are involved in lactate metabolism and contribute to dihydroxylation of bile acids [51]. Indeed, some species metabolize lactate into the short-chain fatty acid (SCFA) acetate and propionate via the methyl-malonyl-CoA pathway [39,51]. However, apart from these few specific findings, given the variability in populations examined and diet assessment tools employed in these studies, their results cannot be collated, and no robust conclusions can be expressed regarding the independent effects of PA/exercise on the gut microbiota of healthy humans.

This review was an attempt to summarize the evidence regarding gut microbial diversity and abundance related to PA, trying to separate the main available evidence from diet contribution, which differentiates our analysis from previous reviews performed on this item.

### 4.3. Limitations

First of all, it should be considered that the majority of the selected studies did not collect lifestyle information through objective measurement tools, such as accelerometers for PA/exercise or a photographic monitoring of dietary intake. Assessing these variables through self-reporting may lead to inaccurate results. Furthermore, separating the effects of diet and PA is difficult to perform, since PA itself can favor the adoption of specific dietary patterns.

### 4.4. Future Research

More randomized controlled studies analyzing wider samples and controlling for potential confounders are needed in this field to disentangle this question. In particular, it should be considered that the practice of sport is often associated with specific and sometimes extreme dietary patterns in professional athletes, same as regular exercise in amateurs is often accompanied by healthier dietary habits [21,23,31,33,38]. In addition, the gut microbiome has been shown to mediate the effect of both diet and exercise, making it relevant to the athletes’ health and performance [52,53]. Thus, it is important that future research implements an appropriate research design to investigate and control for possible confounding effects of diet. Moreover, further studies including the combined analysis of metabolomic and metagenomic data could open new perspectives to investigate diet, sport, and health [53]. Particularly, to optimize microbiota functionality for both athletes and the general population through the design of adequate exercise and dietary programs, the components of the exercise and diet–microbiome paradigm should be further explored.

## Figures and Tables

**Table 1:** Characteristics of the selected studies.

Author, Country, Year	Study Design	Sample Characteristics	Type of PA/Exercise	Exercise Load	Duration of Exercise Intervention	Timing of Assessment	Diet Control/ Assessment	Type of Diet/Nutrients Evaluated	Quality of the Study	References
Barton, UK, 2018	cross-sectional	N = 86 (100% M): 40 elite professional athletes, 46 healthy controls	rugby	/	/	/	assessed (FFQ and photographic food atlas)	total energy and macronutrient intake	JBI: include	[31]
Bressa, Spain, 2017	cross-sectional	N = 40 (100% F): 19 actives, 21 sedentary	physical exercise	active ≥ 3 h of physical exercise per week; sedentary < 3 days of exercise per week for 30 min at a moderate intensity	7 days	1 week	assessed (FFQ)	macronutrients, fiber, ethanol, and main food group intake	JBI: include	[32]
Clarke, USA, 2014	cross-sectional	N = 69 (40 male rugby elite player, 29 male control)	rigorous training in a training camp	/	4 weeks	/	assessed (FFQ and photographic food atlas)	macronutrient, fiber, and supplement intake	JBI: include	[33]
Cronin, Ireland, 2018	randomized controlled trial	N = 90 (41.1% F), aged 18–40 years	aerobic and resistance training	3 times per week moderate aerobic exercise and 7 machine-based resistance exercise	8 weeks	baseline and 8 weeks	assessed (FFQ) controlled (whey protein supplementation)	whey protein supplementation group, whey protein + exercise group, exercise group	CRBT: some concerns	[34]
Gallè, Italy, 2020	cross-sectional study	N = 140 healthy students (17 low active, 57 moderately active, 66 highly active) aged 18–36 years	habitual weekly PA	auto-referred MET-minutes/week	/	/	assessed (questionnaire)	Mediterranean diet adherence	JBI: include	[35]
Han, China, 2020	cross-sectional study	N = 19 healthy female rowing athletes (12 elite and 7 non-elite athletes) aged 12–26 years	rowing	/	Adult elite athletes = 19–26 years (n.6); youth elite athletes = 12–17 years (n.6); youth elite athletes = 12–16 years (n.9)	baseline, from April to May 2017	assessed (FFQ)	drinking, staple food, vegetables, meat poultry, seafood, bean, grease, salt, raw garlic	JBI: include	[36]
Jang, South Korea 2019	cross-sectional	N = 45 male (15 runners,15 bodybuilders and 15 healthy controls)	bodybuilding, running	/	bodybuilding for 7.6 years; running for 7.5 years	/	assessed (food diary + supplements recording)	macronutrient and fiber intake	JBI: include	[37]
Manor, USA, 2020	cross-sectional study	N = 3409 healthy subjects (59% female), mean age 49 ± 12	habitual weekly PA	type, frequency, and duration	/	/	assessed (questionnaire)	food group intake	JBI: include	[38]
Scheiman, USA, 2019	cross-sectional	15 runners and 10 sedentary controls	running	1 marathon	1 day	every day from 1 week before to 1 week after the marathon	assessed (questionnaire + daily annotation sheet)	USDA MyPlate consumption categories, protein powder supplementation	JBI: include	[39]
Taniguchi, Japan, 2018	randomized crossover trial	N = 33 healthy men aged 62–76 years	progressive aerobic exercise	three sessions per week. 60% of pre-exercise VO_2_ peak the first week, 70% during week 2 and 3, 75% week 4 and 5	5 weeks	baseline, week 5 and 10	assessed during the intervention (diet history questionnaire)	food group intake	CRBT: some concerns	[40]

**Table 2:** Main findings related to gut microbiota variability and composition in athletes compared to inactive controls from the selected studies.

Author, Country, Year	Variability	*Firmicutes*	*Bacteroidetes*	*Actinobacteria*	*Proteobacteria*	*Akkermansia*	Synthesis of the Results in Relation with Diet	References
Barton, UK, 2018	↑ Shannon index	↑* (*Erysipelotrichia incertae sedis*)	/	/	/	↑*	Within microbial-derived SCFAs, acetic acid, propionic acid, and butyric acid correlated with fiber and protein, while isobutyric acid, isovaleric acid, and valeric acid correlated with microbial diversity. Significant correlations for targeted measurements of SCFAs were found with *Roseburia* and *Family XIII Incertae Sedis*.	[31]
Clarke, USA 2014	↑ Shannon index ↓ Unifrac distance	↑* *Erysipelotrichia* ↑* *Lactobacillus* ↓* *Ruminococcaceae* ↑* *Dorea*	↑* *Prevotella* ↓* *Bacteroides*	/	/	↑*	The enhanced diversity of the microbiota correlates with exercise and dietary protein consumption in the athlete group.	[33]
Han, China, 2020	↑ Shannon and Simpson index	↑* *Clostridiales* ↑* *Ruminococcaceae* ↑* *Faecalibacterium*	↓*	/	↑*	/	Interperson microbiome variability is mainly affected by dietary factors and physical characteristics.	[36]
Jang, South Korea 2019	↔ beta diversity	↑* *Faecalibacterium* ↑* *Clostridium* (bodybuilders) ↑* *Einsenbergiella* (bodybuilders) ↓* *Blautia* (runners) ↓* *Leuconostoc* (runners) ↓* *Weissella* (runners)	↓* *Bacteroides stercoris* (bodybuilders) ↓*Bacteroides caccae (runners)	↓* *Bifidobacterium* (bodybuilders)	↓* *Parasutterella* (bodybuilders) ↑* *Sutterella* (bodybuilders) ↑* *Haemophilus* (bodybuilders) ↓* *Acinetobacter* (bodybuilders) ↓* *Enterobacter* (runners)	/	Aerobic or resistance exercise training accompanied by an unbalanced intake of macronutrients and low intake of dietary fiber did not lead to increased diversity of gut microbiota; high-protein diets may have a negative impact on gut microbiota diversity for athletes in endurance sports who consume low carbohydrates and low dietary fiber, while athletes in resistance sports that carry out a high-protein–low-carbohydrate and high-fat diet demonstrate a decrease in SCFA-producing commensal bacteria.	[37]
Scheiman, USA, 2019	/	↑ *Veillonella*	/	/	/	/	The observed significance of the association between *Veillonella* relative abundance and pre- and postmarathon state is likely not confounded by any fixed effects.	[39]

↔ no differences between groups; ↓* significant decrease; ↓ nonsignificant decrease; ↑* significant increase; ↑ nonsignificant increase.

**Table 3:** Main findings related to gut microbiota variability and composition in active subjects compared to inactive controls from the selected studies.

Author, Country, Year	Variability	*Firmicutes*	*Bacteroidetes*	*Actinobacteria*	*Proteobacteria*	*Akkermansia*	Synthesis of the Results in Relation with Diet	References
Bressa, Spain, 2017	↔ alpha diversity, beta diversity	↑*	↓* *Barnesiellaceae* ↓ *Odoribacteraceae*	/	/	↑*	Dairy products and cereals were, respectively, positively and negatively related to the abundance of *Turicibacter*; proteins were negatively related to *Bifidobacterium* abundance; diet lipids were positively associated with *Odoribacter* and negatively related to *Ruminococcaceae*; the inverse correlation between fat intake and muscle parameters, and between fiber intake and body fat composition, prevented multiple regression analysis of dietary factors and exercise-related factors together because of collinearity problems.	[32]
Cronin, Ireland, 2018	↑ Shannon index ↔ alpha diversity	/		/	/	/	After the intervention period, bacterial diversity was greater in the exercise–protein-supplementation group than in the protein-supplementation-only group, while the diversity of virus species was lower in the exercise–protein-supplementation group and in the protein-supplementation-only group than in the exercise-only group.	[34]
Gallè, Italy, 2020	↔ Shannon index ↑* beta diversity	↓* *Megasphaera* ↓* *Lachnobacterium* ↓* *Dialister*	↓* *Paraprevotella*	/	/	/	Nor PA level nor diet were significantly associated with the Shannon index and with the F/B ratio.	[35]
Manor, USA, 2020	↑* Shannon index	↑* *Ruminococcaceae* ↑* *Clostridiales* ↑* *Veillonella* ↑* *Lachnospira* ↑* *Faecalibacterium*	/	/	/	/	Associations were tested by fitting linear regression models of Shannon diversity on PA analytes, adjusting for dietary factors. The association with moderate and vigorous activity remained significant.	[38]
Taniguchi, Japan, 2018	↔ alpha diversity	↓* *C. difficile*	/	/	/	/	The nutritional intake was not significantly altered during the exercise intervention; changes in diet during intervention did not seem to influence the results of the study.	[40]

↔ no differences between groups; ↓* significant decrease; ↓ nonsignificant decrease; ↑* significant increase; ↑ nonsignificant increase.

## Data Availability

Data available in a publicly accessible repository.

## Supplementary Materials

The following are available online at https://www.mdpi.com/article/10.3390/nu13061890/s1, Table S1: Risk of bias assessment summary: review authors’ judgements about each methodological quality item for each study included in this review, using the JBI Critical Appraisal Checklist for Analytical Cross-Sectional Studies (2017). Table S2: Risk of bias assessment summary: review authors’ judgements about each methodological quality item for each study included in this review, using the Cochrane Risk of Bias.
