# Supplementary material for: Can Physical Activity Influence Human Gut Microbiota Composition Independently of Diet? A Systematic Review
Source: Nutrients. 2021 May 31;13(6):1890. doi: 10.3390/nu13061890 (PMC8228232; doi:10.3390/nu13061890)
Supplement: Supplementary file 1 [file nutrients-13-01890-s001.zip › nutrients-1204155-supplementary.pdf]

S1 Table. Risk of bias assessment summary: review authors' judgements about each methodological quality item for each study included in this review, using the JBI Critical Appraisal Checklist for Analytical Cross-Sectional Studies (2017) [29].

**Item**	**Barton, UK, 2017 [31]**	**Bressa, Spain, 2017 [32]**	**Clarke, USA, 2014 [33]**	**Gallè, Italy, 2020 [35]**	**Han, China, 2020 [36]**	**Jang, South Korea, 2019 [37]**	**Manor, USA, 2020 [38]**	**Scheiman, USA, 2019 [39]**
1. Were the criteria for inclusion in the sample clearly defined?	Y	Y	Y	Y	Y	Y	Y	Y
2. Were the study subjects and the setting described in detail?	Y	Y	Y	Y	Y	Y	Y	Y
3. Was the exposure measured in a valid and reliable way?	Y	Y	Y	Y	Y	Y	Y	Y
4. Were objective, standard criteria used for measurement of the condition?	Y	Y	Y	Y	Y	Y	Y	Y
5. Were confounding factors identified?	N	Y	Y	Y	Y	Y	Y	N
6. Were strategies to deal with confounding factors stated?	N	Y	Y	Y	Y	Y	Y	N
7. Were the outcomes measured in a valid and reliable way?	Y	Y	Y	Y	Y	Y	Y	Y
8. Was appropriate statistical analysis used?	Y	Y	Y	Y	Y	Y	Y	Y
**Overall appraisal (Included/Excluded)**	**Included**	**Included**	**Included**	**Included**	**Included**	**Included**	**Included**	**Included**
**Y= yes; N=no**								

S2 Table. Risk of bias assessment summary: review authors' judgements about each methodological quality item for each study included in this review, using the Cochrane Risk of Bias Tool for Randomized Controlled Trials [30].

**Item**	**Taniguchi, Japan, 2018 [40]**	**Cronin, Ireland, 2018 [34]**
Domain 1a: Risk of bias arising from the randomization process	N	N
Domain 1b: Risk of bias arising from the timing of identification or recruitment of participants in a cluster-randomized trial	N	N
Domain 2: Risk of bias due to deviations from the intended interventions (effect of adhering to intervention)	N	N
Domain 3: Risk of bias due to missing outcome data	Y	Y
Domain 4: Risk of bias in measurement of the outcome	Y	Y
Domain 5: Risk of bias in selection of the reported result	N	N
**Overall risk-of-bias judgement (Low risk/Some concerns/High risk)**	**Some concerns**	**Some concerns**
**Y= yes; N=no**		
